# Web‐based vestibular rehabilitation in persistent postural‐perceptual dizziness

**DOI:** 10.1002/brb3.2346

**Published:** 2021-09-02

**Authors:** Guttorm Eldøen, Stine E. Kvalheim, Terje Thesen, Åse Mygland, Unn Ljøstad, Siri Bakke, Marit Horsgaard Holo, Ingard Løge, Egil Jonsbu

**Affiliations:** ^1^ Department of Neurology Molde County Hospital More and Romsdal Hospital Trust Molde Norway; ^2^ DPS Solvang Sørlandet Hospital Kristiansand Norway; ^3^ Department of health Faculty of Medicine and Health Science Norwegian University of Science and Technology Trondheim Norway; ^4^ Department of Neurology Sørlandet Hospital Kristiansand Norway; ^5^ Department of Habilitation Sørlandet Hospital Kristiansand Norway; ^6^ Department of Clinical Medicine University of Bergen Bergen Norway; ^7^ Norwegian Online Medical Handbook (NEL) Trondheim Norway; ^8^ Department of Psychiatry, Molde County Hospital More and Romsdal Hospital Trust Molde Norway

**Keywords:** dizziness, exercise therapy, feasibility studies, Internet, neurology, quality of life

## Abstract

**Objectives:**

The aims of the study were to investigate the feasibility and preliminary outcome of a Norwegian web‐based self‐help application for vestibular rehabilitation (VR) among patients with high symptom burden of chronic dizziness fulfilling the criteria for persistent postural‐perceptual dizziness (PPPD).

**Materials and methods:**

The web application consists of six weekly online sessions, with written information and video presentations. It is self‐instructive and freely available on NHI.no (https://nhi.no/for‐helsepersonell/vestibular‐rehabilitering/). Ten consecutive patients referred to a neurologic outpatient clinic for chronic dizziness were included. They signed informed consent forms and were examined at inclusion and after three months. State of health and symptom burden were recorded using Vertigo symptom score (VSS), Niigata symptom score (NPQ), Patient Health Questionnaire (PHQ‐9) and health‐related quality of life score (EQ5D‐5L). Experiences with the program were measured using a semi‐structured interview at the end of the study.

**Results:**

Nine out of ten patients completed the program. The findings suggest that the web application was easy to use, instructive and educatable. Challenges were the load of exercises, motivation to continue training during relapses and performing the body rolling on the floor. Participants had high symptom burden (VSS mean 32.9) and long duration of symptoms in years (mean 11.5). The participants improved on average 6.9 points on the VSS score.

**Conclusions:**

This web application for chronic dizziness appears to be feasible and may reduce symptoms in patients who have struggled with serious and long‐lasting dizziness.

## Abstract 2

The aims of the study were to investigate the feasibility and preliminary outcome of a Norwegian web‐based self‐help application for vestibular rehabilitation (VR) among patients with high symptom burden of chronic dizziness fulfilling the criteria for persistent postural‐perceptual dizziness (PPPD). The findings suggest that the web application was easy to use, instructive, educatable and effective.

## 1 INTRODUCTION

Dizziness is a common condition with 15%–20% of the adult population suffering transient or continuous dizziness each year (Neuhauser, 2016). Persistent postural‐perceptual dizziness (PPPD) is a frequent cause of chronic dizziness, which is possible to treat. It is a newly defined disorder considered to be caused by a long‐term maladaptation to a neuro‐otological, medical or psychological event that triggered vestibular symptoms. Pathophysiologic studies suggest that interactive cortical networks may fail to suppress the bottom‐up influences of instinctive threat on postural control and spatial orientation. PPPD develops in up to 25% of acute vertigo cases or for unknown reasons (Popkirov et al., 2018). The disease is associated with a considerable personal and healthcare burden, such as reduced quality of life, increased sick leave, social withdrawal and high use of healthcare resources (Mueller et al., 2014; Neuhauser et al., 2008; Ruthberg et al., 2020). Anxiety and symptoms of depression are common comorbidities (Dieterich & Staab, 2017; Staab, 2019). In a review study by Kundakci et al., exercise‐based vestibular rehabilitation (VR) shows benefits for adult patients with chronic dizziness with regard to symptom score, fall risk, balance and emotional status (Kundakci et al., 2018). These exercises have been known for many years and have been performed among individuals and in groups (Hall et al., 2016; Kleffelgaard et al., 2016; Popkirov et al., 2018). Despite the evidence of the effectiveness of VR, a survey suggests that <3% of eligible primary care patients with dizziness ever receive VR (Yardley et al., 2004). The Neurological department at Molde county hospital has run courses for such patients alone or in groups for the past 20 years. Web‐based VR is an approach available in English and Dutch versions. Randomized trials have shown that web‐based VR, standing alone or combined with face‐to‐face physiotherapy support, is safe and clinically effective (Geraghty et al., 2017, 2014; van Vugt et al., 2017, 2019, 2020). These RCTs have recruited primary care participants with a relatively low symptom burden (van Vugt et al., 2017; Yardley et al., 2004). The web‐based approach has been promoted as low‐cost, timesaving, of high quality and with a potential to reach large groups of patients.

As far as we know, no studies have evaluated web‐based VR programs given to patients with high symptom burden of dizziness. At Molde Hospital, a Norwegian web application has been developed, completely unguided and available free of charge (Anon, 2020). The purpose of the present study was to investigate the use of this application in a neurological outpatient clinic where the patients suffer long‐lasting and serious forms of PPPD.

The aims were to evaluate the following: the patient's willingness to participate, availability of possible candidates, changes in symptoms of dizziness, functional impact of dizziness, symptoms of depression and quality of life. We also wanted to collect information about the patients’ experiences with the intervention (e.g., whether they found it useful, instructive, educational and acceptable), and their suggestions for changes.

## 2 MATERIALS AND METHODS

### 2.1 Participants

Ten consecutive patients with the diagnosis of PPPD and Vertigo symptom score (VSS) >12 were invited to participate. They had been referred to the outpatient clinic at the department of Neurology in Molde, a small city in western Norway. All accepted the invitation and signed informed consent forms. One dropped out during the study period due to temporary exacerbation of symptoms. All participants had to understand Norwegian and be proficient internet users.

### 2.2 Design

This was a descriptive feasibility study including evaluation of potential change in symptom variables before and after the intervention. The participants were told that the web application was based on recognized principles of VR, created as a web‐based self‐help course. At inclusion a medical examination was performed, and the patients filled in the questionnaires. They received the web‐address and were encouraged to log on at home and start VR following instructions in the web application. The participants were called in for a follow‐up consultation three months after inclusion. One month before this consultation, they received an e‐mail with the same forms of questionnaires as at baseline, and in addition a semi‐structured interview about their experience of participation. At the end of the study, the completed forms were collected, and a medical examination and an interview were performed.

### 2.3 Intervention

The web course lasts for 6 weeks (Anon, 2020). It consists of daily exercises and provides information, written and oral, about chronic dizziness and VR, compensatory mechanisms of the balance system and how these can be improved, as well as various coping strategies. The exercises are shown in video examples. The first lesson starts by showing head exercises and gives information on how to start training. Information and additional exercises are introduced every week for 6 weeks. The exercises begin gently and increased gradually. They consist of head movements (bending backwards and forwards, from shoulder to shoulder and turning to the sides, with open and closed eyes, at rest and while moving the body), eye movements (keeping the eyes fixed in different directions while moving the body), body rolling from side to side in a lying position, head bending toward the floor in a sitting position, standing up from lying to standing position, standing with gathered legs, standing on one leg, standing with heel to toe and walking in a circle, combined with head and eye fixation exercises (Bronstein et al., 2010).

The participants were told to perform the exercises three times a day, starting at a low level, working slowly, and not increasing the load more often than once a week. They were also told that they should experience a mild to moderate degree of dizziness after the exercises, along the way. They could postpone increasing the amount if they thought they were benefiting from the step they were at. The participants were told that they would find all relevant information and instructions in the program (Eldøen et al., 2019). They were encouraged to continue with the exercises beyond the course.

### 2.4 Questionnaires

#### 2.4.1 VSS form

The form consists of 15 questions about symptoms during the last month. Each answer is scored on a 5‐point scale (0–4), and a measure of the severity of the symptoms is obtained by sum‐score. Severe dizziness is defined as ≥12 points on the total scale. The scale includes two subscales: eight selected questions related to vertigo and balance (score ranging from 0 to 32) VSSB, and seven selected questions related to autonomic anxiety symptoms (score ranging from 0 to 28) VSSA. VSS has been widely used in clinical settings and in research in scoring the impact of dizziness and is also translated and validated in Norwegian (Wilhelmsen et al., 2008).

#### 2.4.2 The Niigata persistent positional perceptual dizziness (PPPD) Questionnaire

This form measures symptoms during the last week. It contains 12 questions about three aggravating factors of PPPD (upright posture/gait, movement and visual stimulation). Each factor is evaluated using questions that score the severity from 0 (none) to 6 (unbearable) symptoms. The questionnaire has been translated for the present study, but the Norwegian version has not yet been validated (Yagi et al., 2019).

#### 2.4.3 Patient Health Questionnaire

The Patient Health Questionnaire (PHQ‐9) is a validated nine‐question instrument to screen for symptoms of depression. Scores range from 0 to 27, where a total of 10 or above is suggestive of the presence of depression (Gilbody et al., 2007; Kroenke et al., 2010).

#### 2.4.4 EuroQL 5L‐health‐related quality of life

EuroQL 5L‐health‐related quality of life (EQ5D‐5L) is a standardized generic instrument used to measure health outcomes. The questions are divided into five areas: mobility, self‐care, usual activities, pain/discomfort and anxiety/depression. In addition, EQ VAS measures respondents’ self‐assessed health on a visual analogue scale from 0 to 100, where 0 is defined as the worst imaginable state of health and 100 is the best imaginable state of health. This can be used as a quantitative measure of health outcomes that reflects the patient's own assessment. In the present study, only the EQ VAS is presented (Janssen et al., 2013).

#### 2.4.5 Semi‐structured interview

We developed a semi‐structured (conversation) interview for the study. It consisted of six questions, rated 0–6 from bad to very good, on whether the program worked well, if it included the right amount of exercises, if the text was understandable and instructive, what benefit there was and the degree of subjective improvement. Participants were also asked for suggestions for improvement.

#### 2.4.6 Ethics

The participants were informed that participation was voluntary and that they could withdraw from the study at any time. Participants were free to use the healthcare system in their usual way, if necessary. Basically, we perceive the exercises in the VR‐course as harmless (van Vugt et al., 2019). If the participants could not endure the exercises, they were advised to quit and contact the healthcare system or the study organizers.

Trial registration: NCT04458376. The study was approved by the Regional Committees for Medical and Health Research Ethics (REC):137107.

### 2.5 Statistics

Mean scores and corresponding standard deviation (SD) are presented for both baseline and follow‐up. In addition, we present mean difference with 95% confidence interval (Cl). The Statistical Package for Social Sciences (SPSS) version 27 was used for all analyses.

## 3 RESULTS

The participants were women, aged from 19 to 82 years (mean 49), symptom duration from 1.5 to 54 years (mean 11.5) and with a symptom score of VSS from 17 to 53 (mean 32.9). Three participants had anxiety, depression or social phobia. Four had migraines, three had tension headaches, two had ringing in the ears and hearing loss and two had cardiovascular disorder and were taking beta‐blockers and blood pressure medication. One used benzodiazepine. All of them had at least one MRI of the brain taken. MRI/CT were normal in all participants and had been performed a total of 30 times for the whole group in the last 15 years, according to hospital journals, with an average of 3.3 times per participant. All of them were highly willing to participate and were motivated for the treatment. One participant was given sick leave by the doctor for a period of time to complete the exercises. She thought that this was her last chance of recovery, and she improved in VSS (from 38 to 17). She decided to continue the exercises in the months ahead.

### 3.1 Improvement in symptoms and function score

Seven of nine participants improved in VSS, and the mean scores are present in Table 1.

**TABLE 1:** Scores reported by the participants at baseline and after three months

Score forms	Baseline mean score (SD)	Follow‐up after 3 months Mean score (SD)	Mean difference (95% CI)
VSS	32.9 (11.2)	26.0 (11.1)	6.9 (0.2–13.6)
VSSB	21.7 (5.9)	16.8 (6.8)	4.9 (−10.8 to 1.0)
VSSA	11.2 (8.0)	9.2 (7.2)	2 (−7.7 to 3.7)
NPQ	37.3 (10.9)	37.1 (15.0)	0.2 (−8.2 to 7.8)
PHQ‐9	9.6 (5.1)	10.2 (6.0)	−0.7 (−2.3 to 3.6)
EQ5D‐5L	62.5 (15.2)	57.5 (22.1)	5.0 (−1.5 to 3.2)

Abbreviations: CI, confidence internal; EQ5D‐5L, EuroQL 5L‐health‐related quality of life; NPQ, The Niigata persistent positional perceptual dizziness (PPPD) Questionnaire; PHQ‐9, Patient Health Questionnaire; VSS, Vertigo symptom score; VSSB, Vertigo symptom score form balance; VSSA, Vertigo symptom score form anxiety;.

#### 3.1.1 Patients’ experience of the application

The participants reported that the web application was easily accessible (mean score 4.7 [range 3–5]), understandable (mean 3.9 [range 2–5]), worked well (mean 2.9 [range 1–5]), had the right amount of exercises (mean 2.8 [range 0–5]), “ I had great benefit” (mean 2.3 [range 0–5]) and “I made great progress” (mean 2.2 [range 0–5]). In free‐text, they reported that the sessions were easy to follow and understand and were acceptable. Some of the participants had been struggling to find the right amount of intensity and dosage of the exercises. They found that their naturally fluctuating course of symptoms made exercise challenging during deterioration. Some failed to carry out the exercises of rolling around on the floor and suggested that exercise should be optional. In general, they reported that they were highly motivated, but it was not easy to find time during the day.

### 3.2 Adverse events

No serious incidents were reported throughout the course.

## 4 DISCUSSION

This study is the first evaluation of an unguided web‐based VR‐program given to patients requisitioned from a hospital outpatient clinic, with long‐lasting and very high symptom burden, with age over 18 years. The patients’ willingness to participate was high. The web application appears to have high availability, and the participants showed improvements in their dizziness. They were satisfied with the design of the web application, and it was easy to use, understandable and instructive. The majority were satisfied with their own achievements and progress, but some of them found performing all the exercises a challenge.

All invited patients agreed to participate. This corresponds to the clinical impression that this patient group suffers a high symptom burden and shows extensive willingness to seek improvement. They appear to be interested in practicing an exercise program instead of taking medication. They describe the program as demanding, but at the same time only one participant dropped out during the three months. This high degree of acceptability corresponds well to a similar study (van Vugt et al., 2017).

There was a wide range of both age and symptom duration among the participants in this study, and they all had a high symptom burden. They tolerated the program well and as a group the symptoms improved. This indicates that the program is suitable for many patients suffering from PPPD. The findings are consistent with those of van Vugt et al., who conclude that VR is a good tool for dizzy patients even when no definite causal etiology has been made (van Vugt et al., 2017).

The burden of dizziness improved in seven out of nine participants. The studies from van Vugt et al. and Geraghty et al. show a VSS baseline score about 14, while our VSS baseline mean is 32.9. The VSS threshold (>12) suggests “severe dizziness” in other studies (Fox et al., 2019; Wilhelmsen et al., 2008). The baseline score of PHQ‐9 is about 5 in the study from van Vugt et al. compared to 10 in the present study. A total of 10 points in PHQ‐9 or above are suggestive of the presence of depression (Gilbody et al., 2007). This difference in these study populations indicates that our participants had a significantly higher burden of disease. The mean score of improvement in VSS in the present study is comparable with other studies (van Vugt et al., 2017), indicating that the program works well for unselected patients with a high disease burden. However, the result must be interpreted with caution as this is a small study with an uncontrolled design.

We did not find any significant change in depressive symptoms or health‐related quality of life for the whole group. The lack of reported improvements is in line with previous studies, where additional support is often needed to affect these variables (Apolinario‐Hagen, 2019; Cuijpers et al., 2017). Specific cognitive therapy and medications may have a role in the management of dizziness where comorbidity with mental disorders is present (Axer et al., 2020; Ketola et al., 2015; Staab, 2019).

We did not measure physical activity, but some participants reported that they had become more active. This may be one explanation of the lack of improvement in the functional score related to dizziness (Niigata PPPD Questionnaire), which focused on symptoms in upright posture/gait, movement and visual stimulation.

The feedback from the semi‐structured interview shows that the training is demanding, and it can be challenging to set aside time in everyday life. The participants need high motivation to complete the program. It could also be difficult to find the right amount of exercises. Participants reported the lowest score (mean 2.2 [range 0–5]) when they rated their own improvement. This finding probably reflects both large differences among the participants and that some had high expectations. Participants stated that it would be feasible to continue beyond the three months in the study (Ketola et al., 2015; Staab, 2019). The natural course of PPPD is wide fluctuations in symptoms over days and during a day. Participants with a heavy burden of disease struggle to find motivation for training when meeting relapses. The only suggestion for change to the program was that the exercises of rolling around on the floor should be optional. We agree that this should be mentioned in the program.

### 4.1 Strengths and limitations

The strength of this study is that the participants represent a wide age distribution, they were motivated and had a high burden of disease with long duration of symptoms. The participants were representative for the main goal of the study in examining whether the web application is feasible. It is a caveat in relation to scoring results that disease burden is influenced by the daily variations of symptoms. The limitations are a low number of participants, lack of control group, overrepresentation of women and a short observation time for assessing the consistency of improvement.

### 4.2 Implications for clinical practice and further research

Patients with chronic dizziness (PPPD) and a high symptom burden may be introduced to VR. A web‐based self‐help application of this type is feasible. The results of the present study open an opportunity to offer guided web‐based VR also to patients with high disease burden and long‐lasting symptoms.

This field needs more studies both in determining who is best suited and whether the improvement lasts over time. Recording change in physical activity should be considered. A broader survey of health status, activity and psycho‐social data could have made it easier to evaluate the results.

## 5 CONCLUSION

The Norwegian version of a self‐help web application for VR appears to be a feasible and possibly effective treatment for a long‐lasting and high‐symptom burden.

### TRANSPARENT PEER REVIEW

The peer review history for this article is available at https://publons.com/publon/10.1002/brb3.2346

## Data Availability

Due to the nature of this research, participants of this study did not agree for their data to be shared publicly, so supporting data is not available.

## References

[brb32346-bib-0001] Anon (2020). *Norsk elektronisk legehåndbok (NHI.no)*. https://nhi.no/for‐helsepersonell/vestibular‐rehabilitering/

[brb32346-bib-0002] Apolinario‐Hagen, J. (2019). Internet‐delivered psychological treatment options for panic disorder: A review on their efficacy and acceptability. Psychiatry Investigation, 16(1), 37–49. 10.30773/pi.2018.06.26 30122031 PMC6354039

[brb32346-bib-0003] Axer, H. , Finn, S. , Wassermann, A. , Guntinas‐Lichius, O. , Klingner, C. M. , & Witte, O. W. (2020). Multimodal treatment of persistent postural‐perceptual dizziness. Brain and Behavior, 10(12), e01864. 10.1002/brb3.1864 32989916 PMC7749543

[brb32346-bib-0004] Bronstein, A. M. , Lempert T. , & Seemungal B. M. (2010). Chronic dizziness: A practical approach. Practical Neurology, 10(3), 129–139. 10.1136/jnnp.2010.211607 20498184

[brb32346-bib-0005] Cuijpers, P. , Kleiboer, A. , Karyotaki, E. , & Riper, H. (2017). Internet and mobile interventions for depression: Opportunities and challenges. Depression and Anxiety, 34(7), 596–602. 10.1002/da.22641 28471479

[brb32346-bib-0006] Dieterich, M ., & Staab J. P. (2017). Functional dizziness: From phobic postural vertigo and chronic subjective dizziness to persistent postural‐perceptual dizziness. Current Opinion in Neurology, 30(1), 107–113. 10.1097/WCO.0000000000000417 28002123

[brb32346-bib-0007] Eldøen, G. , Ljøstad, U. , Goplen, F. K. , Aamodt, A. H. , & Mygland, Å. (2019). Persistent postural‐perceptual dizziness. Tidsskrift for Den Norske Laegeforening, 139(9). 10.4045/tidsskr.18.0962 31140261

[brb32346-bib-0008] Fox, A. , Riska, K. , Tseng, C. L. , McCarron, K. , Satcher, S. , & Osinubi, O. (2019). Helmer, dizziness, vertigo, and mental health comorbidity in Gulf War veterans. Journal of the American Academy of Audiology, 30(9), 764–771. 30424834 10.3766/jaaa.17122

[brb32346-bib-0009] Geraghty, A. W. A. , Essery, R. , Kirby, S. , Stuart, B. , Turner, D. , Little, P. , Bronstein, A. , Andersson, G. , Carlbring, P. , & Yardley, L. (2017). Internet‐based vestibular rehabilitation for older adults with chronic dizziness: A randomized controlled trial in primary care. Annals of Family Medicine, 15(3), 209–216. 10.1370/afm.2070 28483885 PMC5422081

[brb32346-bib-0010] Geraghty, A. W. , Kirby, S. , Essery, R. , Little, P. , Bronstein, A. , Turner, D. , Stuart, B. , Andersson, G. , Carlbring, P. , & Yardley, L. (2014). Internet‐based vestibular rehabilitation for adults aged 50 years and over: A protocol for a randomised controlled trial. BMJ Open, 4(7), e005871. 10.1136/bmjopen-2014-005871 PMC4120362 25052178

[brb32346-bib-0011] Gilbody, S. , Richards, D. , Brealey, S. , & Hewitt, C. (2007). Screening for depression in medical settings with the Patient Health Questionnaire (PHQ): A diagnostic meta‐analysis. Journal of General Internal Medicine, 22(11), 1596–1602. 10.1007/s11606-007-0333-y 17874169 PMC2219806

[brb32346-bib-0012] Hall, C. D. , Herdman, S. J. , Whitney, S. L. , Cass, S. P. , Clendaniel, R. A. , Fife, T. D. , Furman, J. M. , Getchius, T. S. , Goebel, J. A. , Shepard, N. T. , & Woodhouse, S. N. (2016). Treatment for vestibular disorders: How does your physical therapist treat dizziness related to vestibular problems? Journal of Neurologic Physical Therapy, 40(2), 156. 10.1097/01.NPT.0000481898.55592.6e 26934418 PMC4795095

[brb32346-bib-0013] Janssen, M. F. , Pickard, A. S. , Golicki, D. , Gudex, C. , Niewada, M. , Scalone, L. , Swinburn, P. , & Busschbach, J. (2013). Measurement properties of the EQ‐5D‐5L compared to the EQ‐5D‐3L across eight patient groups: A multi‐country study. Quality of Life Research, 22(7), 1717–1727. 10.1007/s11136-012-0322-4 23184421 PMC3764313

[brb32346-bib-0014] Ketola, S. , Havia, M. , Appelberg, B. , & Kentala, E. (2015). Psychiatric symptoms in vertiginous patients. Nordic Journal of Psychiatry, 69(4), 287–291. 10.3109/08039488.2014.972976 25394373

[brb32346-bib-0015] Kleffelgaard, I. , Soberg, H. L. , Bruusgaard, K. A. , Tamber, A. L. , & Langhammer, B. (2016). Vestibular rehabilitation after traumatic brain injury: Case series. Physical Therapy, 96(6), 839–849. 10.2522/ptj.20150095 26586860

[brb32346-bib-0016] Kroenke, K. , Spitzer, R. L. , Williams, J. B. , & Löwe, B. (2010). The patient health questionnaire somatic, anxiety, and depressive symptom scales: A systematic review. General Hospital Psychiatry, 32(4), 345–359. 10.1016/j.genhosppsych.2010.03.006 20633738

[brb32346-bib-0017] Kundakci, B. , Sultana, A. , Taylor, A. J. , & Alshehri, M. A. (2018). The effectiveness of exercise‐based vestibular rehabilitation in adult patients with chronic dizziness: A systematic review. F1000Re search, 7, 276. 10.12688/f1000research.14089.1 PMC5954334 29862019

[brb32346-bib-0018] Mueller, M. , Strobl, R. , Jahn, K. , Linkohr, B. , Peters, A. , & Grill, E. (2014). Burden of disability attributable to vertigo and dizziness in the aged: Results from the KORA‐Age study. European Journal of Public Health, 24(5), 802–807. 10.1093/eurpub/ckt171 24213583

[brb32346-bib-0019] Neuhauser, H. K. (2016). The epidemiology of dizziness and vertigo. Handbook of Clinical Neurology, 137, 67–82. 10.1016/B978-0-444-63437-5.00005-4 27638063

[brb32346-bib-0020] Neuhauser, H. K. , Radtke, A. , von Brevern, M. , Lezius, F. , Feldmann, M. , & Lempert, T. (2008). Burden of dizziness and vertigo in the community. Archives of Internal Medicine, 168(19), 2118–2124. 10.1001/archinte.168.19.2118 18955641

[brb32346-bib-0021] Popkirov, S. , Stone, J. , & Holle‐Lee, D. (2018). Treatment of persistent postural‐perceptual dizziness (PPPD) and related disorders. Current Treatment Options in Neurology, 20(12), 50. 10.1007/s11940-018-0535-0 30315375

[brb32346-bib-0022] Ruthberg, J. S. , Rasendran, C. , Kocharyan, A. , Mowry, S. E. , & Otteson, T. D. (2020). The economic burden of vertigo and dizziness in the United States. Journal of Vestibular Research, 31(2), 81–90. 10.3233/VES-201531 33285661

[brb32346-bib-0023] Staab, J. P. (2019). Psychiatric considerations in the management of dizzy patients. Advances in Oto‐Rhino‐Laryngology, 82, 170–179. 30947209 10.1159/000490286

[brb32346-bib-0024] van Vugt, V. A. , Heymans, M. W. , van der Wouden, J. C. , van der Horst, H. E. , & Maarsingh, O. R. (2020). Treatment success of internet‐based vestibular rehabilitation in general practice: Development and internal validation of a prediction model. BMJ Open, 10(10), e038649. 10.1136/bmjopen-2020-038649 PMC7569931 33067287

[brb32346-bib-0025] van Vugt, V. A. , van der Wouden, J. C. , Bosmans, J. E. , Smalbrugge, M. , van Diest, W. , Essery, R. , Yardley, L. , van der Horst, H. E. , & Maarsingh, O. R. (2017). Guided and unguided internet‐based vestibular rehabilitation versus usual care for dizzy adults of 50 years and older: A protocol for a three‐armed randomised trial. BMJ Open, 7(1), e015479. 10.1136/bmjopen-2016-015479 PMC5253547 28110290

[brb32346-bib-0026] van Vugt, V. A. , van der Wouden, J. C. , Essery, R. , Yardley, L. , Twisk, J. W. R. , van der Horst, H. E. , & Maarsingh, O. R. (2019). Internet based vestibular rehabilitation with and without physiotherapy support for adults aged 50 and older with a chronic vestibular syndrome in general practice: Three armed randomised controlled trial. BMJ Clinical Research, 367, l5922. 10.1136/bmj.l5922 PMC6829201 31690561

[brb32346-bib-0027] Wilhelmsen, K. , Strand, L. I. , Nordahl, S. H. G. , Eide, G. E. , & Ljunggren, A. E. (2008). Psychometric properties of the Vertigo symptom scale—Short form. BMC Ear Nose Throat Disord, 8, 2. 10.1186/1472-6815-8-2 PMC2329601 18371190

[brb32346-bib-0028] Yagi, C. , Morita, Y. , Kitazawa, M. , Nonomura, Y. , Yamagishi, T. , Ohshima, S. , Izumi, S. , Takahashi, K. , & Horii, A. (2019). A validated questionnaire to assess the severity of persistent postural‐perceptual dizziness (PPPD): The Niigata PPPD Questionnaire (NPQ). Otology & Neurotology, 40(7), e747–e752. 10.1097/MAO.0000000000002325 31219964 PMC6641087

[brb32346-bib-0029] Yardley, L. , Donovan‐Hall, M. , Smith, H. E. , Walsh, B. M. , Mullee, M. , & Bronstein, A. M. (2004). Effectiveness of primary care‐based vestibular rehabilitation for chronic dizziness. Annals of Internal Medicine, 141(8), 598–605. 10.7326/0003-4819-141-8-200410190-00007 15492339

